# Robust spectral analysis of videocapsule images acquired from celiac disease patients

**DOI:** 10.1186/1475-925X-10-78

**Published:** 2011-09-09

**Authors:** Edward J Ciaccio, Christina A Tennyson, Govind Bhagat, Suzanne K Lewis, Peter HR Green

**Affiliations:** 1 Department of Medicine - Celiac Disease Center, Columbia University, New York, USA; 2 Department of Pathology and Cell Biology, Columbia University, New York, USA

**Keywords:** celiac disease, ensemble average, Fourier transform, small intestine, spectral analysis

## Abstract

**Background:**

Dominant frequency (DF) analysis of videocapsule endoscopy images is a new method to detect small intestinal periodicities that may result from mechanical rhythms such as peristalsis. Longer periodicity is related to greater image texture at areas of villous atrophy in celiac disease. However, extraneous features and spatiotemporal phase shift may mask DF rhythms.

**Method:**

The robustness of Fourier and ensemble averaging spectral analysis to compute DF was tested. Videocapsule images from the distal duodenum of 11 celiac patients (frame rate 2/s and pixel resolution 576 × 576) were analyzed. For patients 1, 2, ... 11, respectively, a total of 10, 11, ..., 20 sequential images were extracted from a randomly selected time epoch. Each image sequence was artificially repeated to 200 frames, simulating periodicities of 0.2, 0.18, ..., 0.1Hz, respectively. Random white noise at four different levels, spatiotemporal phase shift, and frames with air bubbles were added. Power spectra were constructed pixel-wise over 200 frames, and an average spectrum was computed from the 576 × 576 individual spectra. The largest spectral peak in the average spectrum was the estimated DF. Error was defined as the absolute difference between actual DF and estimated DF.

**Results:**

For Fourier analysis, the mean absolute error between estimated and actual DF was 0.032 ± 0.052Hz. Error increased with greater degree of random noise imposed. In contrast, all ensemble average estimates precisely predicted the simulated DF.

**Conclusions:**

The ensemble average DF estimate of videocapsule images with simulated periodicity is robust to noise and spatiotemporal phase shift as compared with Fourier analysis. Accurate estimation of DF eliminates the need to impose complex masking, extraction, and/or corrective preprocessing measures.

## Background

Celiac disease is an autoimmune disease which often manifests as villous atrophy in the small intestinal lining or mucosa [1-3]. The result can be fissuring and mosaic pattern of the mucosal surface and scalloped appearance of the small intestinal mucosal folds, which result in textural changes that are often visually evident in the acquired videocapsule images. During quantitative analysis, extraneous features including air bubbles and opaque fluids in the small intestinal lumen, as well as random imaging noise, are ubiquitous in videocapsule data [4-6]. These distorting factors can in part be remediated by their delineation followed by masking or extraction; however such systems can be complex, computationally unwieldy, and their sensitivity is somewhat modest [7]. Gastrointestinal motility can be altered in untreated celiac disease [1-3], suggesting the possibility that the frequency content of frame-to-frame changes in videocapsule image texture might vary in these patients. Longer periodicity in the frequency spectra would possibly be indicative of slower motility, while shorter periodicity may suggest faster motility [8]. To validate such measurements, it should be determined whether known, periodic changes in videocapsule image frames, in the presence of image degradation, can be detected using methods of spectral analysis.

The dominant frequency (DF) is useful to estimate the electrical activation rate of excitable media [9,10]. It is defined as the fundamental frequency with greatest spectral power in the physiologic range of interest. The standard method for detecting the DF includes a preprocessing step in which the signal is bandpass filtered, followed by signal rectification, and finally by low pass filtering. However, this manipulation distorts the signal and can cause error in DF estimation [11-13]. Furthermore, it is not very robust to phase and random noise [14,15]. Optimization of filter parameters can improve DF estimation by enabling more spectral power associated with periodic components to pass through the filter [15]. Alternatively, a new method based on ensemble averaging can be utilized to estimate DF [14,15]. This new method does not distort the signal prior to quantitation, because preprocessing measures are not imposed.

In this study, spectral analysis of videocapsule image series was used to determine whether synthesized periodicity could be accurately detected in the presence of phase and random noise, and when extraneous features are imposed. Validation would suggest that spectral analysis of videocapsule image series can be used to detect repetitive patterns in gastrointestinal motility that are potentially important to diagnosis and treatment of celiac disease and other gastrointestinal diseases associated with small intestinal lesions.

## Methods

### A. Clinical Procedure and Data Acquisition

All patients were evaluated at Columbia University Medical Center, New York, from May 1, 2008 to July 31, 2009. Retrospective videocapsule endoscopy data was obtained from 11 celiac patients on a regular diet or within a few weeks of starting a gluten-free diet. In these patients the diagnostic biopsy, taken while on a regular diet, showed Marsh grade II-IIIC lesions. Informed consent was obtained prior to videocapsule endoscopy. Indications for this procedure included suspected celiac disease or Crohn's disease, iron deficiency anemia, obscure bleeding, and chronic diarrhea. All patients had serology- proven and biopsy-proven celiac disease. These patients were subsequently evaluated by videocapsule endoscopy because they were considered to have complicated disease with symptoms such as abdominal pain unexplained by previous evaluation. Excluded were patients under 18 years of age, those with a history of or suspected small bowel obstruction, dysphagia, presence of pacemaker or other electromedical implants, previous gastric or bowel surgery, serum IgA deficiency, pregnancy, and nonsteroidal anti-inflammatory drug (NSAID) use during the previous month. Only complete videocapsule endoscopy studies, reaching the colon, were used for analysis. The retrospective analysis of videocapsule endoscopy data was approved by the Internal Review Board at Columbia University Medical Center.

The PillCamSB2 videocapsule (Given Imaging, Yoqneam, Israel) was utilized to obtain the small bowel images in the study groups [16]. The system consists of a recorder unit with cradle and harness, battery pack and charger, antenna lead set, and real-time viewer. The capsule acquires two digital image frames per second and is a single-use pill-sized device [16]. For each patient undergoing the procedure, abdominal leads were placed on the upper, mid, and lower abdomen, and a belt containing the data recorder and battery pack was strapped about the waist. All subjects swallowed the videocapsule with radio transmitter in the early morning with approximately 0.2 liters of water after a nighttime fast without bowel preparation. Subjects were allowed to drink water 2 hours after ingesting the capsule, and to eat a light meal after 4 hours. The recorder received the radioed images that were transmitted by the videocapsule as it passed through the gastrointestinal tract. The capsule reached the cecum in all participants from which retrospective data was used in this study. The belt data recorder was then removed, and the data was downloaded to a HIPAA-compliant PC-based computer console with proprietary software. Videos were reviewed and interpreted by three experienced gastroenterologists. Videoclips to be used for further quantitative analysis were then exported to external media without patient identifiers. The videoclips were 200 frames each and were acquired from the distal duodenum.

### B. Creation of Synthetic Sequences for Analysis

From each RGB color videoclip, grayscale images (256 brightness levels, 0 = black, 255 = white) with an image resolution of 576 × 576 pixels, were extracted using Matlab Ver. 7.7, 2008 (The MathWorks, Natick MA). One sequence of frames was extracted from each videoclip per patient. Table 1 shows the relationships. The patient number is noted in the left-hand column. The number of image frames extracted from each videoclip for this patient is given in the middle column. These frames were repeated to form a 200 frame sequence. Thus the total number of repeating sequences, sequence length = w, is: 

**Table 1:** Simulated Periodicity from Patient Data

Patient	# frames w	frequency(Hz)
1	10	0.2

2	11	0.182

3	12	0.166

4	13	0.154

5	14	0.142

6	15	0.134

7	16	0.126

8	17	0.118

9	18	0.112

10	19	0.106

11	20	0.1

(1) n=200∕w

The simulated frequencies of these sequences, shown in the right column, was calculated using:

(2) $$\begin{array}{ccc} \text{f} & =\text{sample}\;\text{rate}∕\text{w} & \\ & =2.∕\text{w} \end{array}$$

### C. Synthetic Sequence Degradation

Each of the synthetic image sequences was corrupted using the following techniques:

1. Temporal phase noise: the 200 frame series was altered by removing up to 4 frames from the beginning or end of one of the repeating series comprising the sequence, and appending it to another series. This was done three times at random in each 200 frame series. For example, suppose the period of a sequence *S *is 10 image frames:

(3) $$S\;=\;{\text{M}}_{1},{\text{M}}_{2},{\text{M}}_{3},....\;{\text{M}}_{10},{\text{M}}_{1},{\text{M}}_{2},{\text{M}}_{3},....\;{\text{M}}_{10},....\;$$

where *S *is comprised of 200 total images **M**, and the bold font in **M **denotes that it is a matrix with dimension 576 × 576 pixels. Thus the series of 10 images are repeated 20 times to form a set of 200 images. An example of temporal phase shift by ± 2 that would create an altered sequence *S' *is:

(4) $$\begin{array}{c} S^{\prime }\;=\;{\text{M}}_{3},{\text{M}}_{4},{\text{M}}_{5},{\text{M}}_{6},....\;{\text{M}}_{10},\;{\text{M}}_{1},{\text{M}}_{2},{\text{M}}_{3},....\;{\text{M}}_{10},\;{\text{M}}_{1},{\text{M}}_{2},{\text{M}}_{1},{\text{M}}_{2},{\text{M}}_{3},....\;{\text{M}}_{10},\;{\text{M}}_{1},{\text{M}}_{2},\\ {\text{M}}_{3},....\;{\text{M}}_{10},\;{\text{M}}_{3},{\text{M}}_{4},{\text{M}}_{5},{\text{M}}_{6},....\;{\text{M}}_{10},\;{\text{M}}_{1},{\text{M}}_{2},{\text{M}}_{3},....\;{\text{M}}_{10},\;{\text{M}}_{1},{\text{M}}_{2},{\text{M}}_{3},....\;{\text{M}}_{10},\;{\text{M}}_{1},{\text{M}}_{2},\;{\text{M}}_{1},{\text{M}}_{2},\\ {\text{M}}_{3},....\;{\text{M}}_{10},{\text{M}}_{1},{\text{M}}_{2},{\text{M}}_{3},....\;{\text{M}}_{10},{\text{M}}_{1},{\text{M}}_{2},{\text{M}}_{3},....\;{\text{M}}_{10},{\text{M}}_{2},{\text{M}}_{3},{\text{M}}_{4},{\text{M}}_{5},{\text{M}}_{6},....\;{\text{M}}_{10},{\text{M}}_{1},{\text{M}}_{2},\\ {\text{M}}_{3},....\;{\text{M}}_{10},{\text{M}}_{1},{\text{M}}_{2},{\text{M}}_{3},....\;{\text{M}}_{10},{\text{M}}_{1},{\text{M}}_{1},{\text{M}}_{2},{\text{M}}_{3},....\;{\text{M}}_{10},{\text{M}}_{1},{\text{M}}_{2},{\text{M}}_{3},....\;{\text{M}}_{10},\ldots \end{array}$$

where phase shifts of one or two images occurred during periods 1-3, 5-8, and 11-14 in Eq. 4.

2. Spatial phase noise: each image in the 200 frame series was altered using a row-by-row pixel rotation of up to 20 pixels. The degree of pixel rotation was the same for each row in a particular image, but was varied randomly, in the range 0 to 20, from one image to the next. Thus for example, the original image frame can be written as:

(5) $$\text{M}=\left[\begin{array}{c} \underset{}{\text{m}}\left(1:576\right)\\ \underset{}{\text{m}}\left(1:576\right)\\ \cdots \\ \underset{}{\text{m}}\left(1:576\right) \end{array}\right]$$

where m is a row vector of dimension 576 and the matrix **M **has 576 columns. If the row vectors are phase shifted by +5, then the spatially phase shifted image **M' **is given by:

(6) $${\text{M}}^{\prime }=\left[\begin{array}{c} \underset{}{\text{m}}\left(6:576,\;1:5\right)\\ \underset{}{\text{m}}\left(6:576,\;1:5\right)\\ \cdots \\ \underset{}{\text{m}}\left(6:576,\;1:5\right) \end{array}\right]$$

3. Addition of random white noise: a series of × image frames were removed from the end of the 200 frame series and replaced with × white noise frames, where the number of frames removed was varied from × = 0 frames (0%), 50 frames (25%), 100 frames (50%), 150 frames (75%), and 180 frames (90%). For example for sequence *S *with periodicity = 10, and when 180 noise frames (90%) are substituted into the sequence, then the altered sequence *S' *is in this case:

(7) $$S^{\prime }={\text{M}}_{1},{\text{M}}_{2},{\text{M}}_{3},\ldots .\;{\text{M}}_{10},{\text{M}}_{1},{\text{M}}_{2},{\text{M}}_{3},\ldots \;{\text{M}}_{10},{\text{N}}_{1},{\text{N}}_{2},{\text{N}}_{3},\ldots {\text{N}}_{180}$$

where each matrix **N **is a random noise frame different from all other noise frames.

4. Addition of air bubbles: seven images were randomly removed from the 200 frame series and replaced with an image **B **composed primarily of air bubbles that did not belong in the series. An example of such an altered sequence *S' *is:

(8) $$\begin{array}{c} S^{\prime }\;=\;{\text{M}}_{1},{\text{M}}_{2},{\text{M}}_{3},{\text{M}}_{4},\text{B},\ldots .\;{\text{M}}_{10},{\text{M}}_{1},{\text{M}}_{2},\text{B},\ldots .\;{\text{M}}_{10},{\text{M}}_{1},{\text{M}}_{2},{\text{M}}_{3},\text{B},\ldots .\;{\text{M}}_{10},\ldots .{\text{M}}_{1},\\ {\text{M}}_{2},{\text{M}}_{3},\ldots .\;{\text{M}}_{10},{\text{M}}_{1},{\text{M}}_{2},{\text{M}}_{3},{\text{M}}_{4},{\text{M}}_{5},{\text{M}}_{6},\text{B},\ldots .\;{\text{M}}_{10},{\text{M}}_{1},\text{B},{\text{M}}_{3},\ldots .{\text{M}}_{10},\ldots {\text{M}}_{1},{\text{M}}_{2},{\text{M}}_{3},\ldots .\;\\ {\text{M}}_{10},\text{B},{\text{M}}_{2},{\text{M}}_{3}\ldots .\;{\text{M}}_{9},\text{B},{\text{M}}_{1},{\text{M}}_{2},{\text{M}}_{3}\ldots .\;{\text{M}}_{10},\ldots . \end{array}$$

where frames **B **containing extraneous air bubbles are substituted at random into the 200 frame sequence.

Frequency spectra were calculated for each 200 frame sequence using the methods of sequence degradation as listed above. The four different levels of random white noise frames were added in separate trials.

### D. Spectral Analysis

Both the Fourier and ensemble power spectral methods were used for frequency calculation [9-15,17]. For analysis, each pixel location in the sequence of 200 images was treated as a distinct signal. Each of these 576 × 576 = 331776 signals was first set to mean zero. Then the power spectrum (Fourier or ensemble method) was computed for each, and the average frequency spectrum computed by summing the 331776 individual power spectra and dividing by 331776 was used for further analysis. The tallest peak in this spectrum was considered to be the dominant frequency. In accord with another study [8], these measurements can alternatively be expressed by the dominant period (DP), which is defined as:

(9) DP=2.∕DF

where 2./s is the frame rate, the units of DF are Hz, and the units of DP are seconds.

All calculations were done on a PC-type laptop computer using the Intel Visual Fortran Compiler 9.0 Build Environment for 32-bit applications (Intel Corporation, Santa Clara, California, 2005). For Fourier spectral calculation, the 200 point data array was first smoothed using a Hann window. The windowed data was then padded with zeros to form an array 1024 points to achieve a final resolution of 2./1024 = 0.002Hz. The Fast Fourier Transform (FFT) was computed using subroutine 'four1' provided by Numerical Recipes in Fortran 77 [18], and the power spectrum was generated from the magnitude of the real and imaginary parts of the FFT components, and plotted versus frequency. This radix-2 implementation, which is applicable to real data arrays of length 2^N^, is widely used in the literature, although it is not the most efficient FFT code [19]. In a previous gastrointestinal study, the DP has been observed in the range 1-20 seconds [8]. Thus for Fourier analysis, the spectral range to detect the DF/DP was selected as 0.05Hz (period = 20 seconds) to 0.67 Hz (period ~ 1 second). This implementation did not include preprocessing by bandpass filtering, lowpass filtering, and rectification.

Ensemble average spectral analysis was also coded in Fortran, and has been described in detail elsewhere [8,14,15,17]. Briefly, the mean level is removed from each 200 pixel sequence as for Fourier analysis. As for Fourier analysis, ensemble averages are computed from 0.05Hz (periodicity of w = 40 frames) to 0.67Hz (periodicity of w = 3 frames). For simplicity, calculations were only done at integer values of w; thus only 38 points were computed for ensemble spectral analysis (average resolution of 0.62Hz/38 = 0.016Hz). For any particular pixel trace p_ij_, i = 1,576 and j = 1,576, from 1 to 200 frames, the ensemble average is:

(10) $${\underline{\text{e}}}_{\text{ij}}\left(\text{w}\right)=1∕\text{n}\cdot \left[{\underline{\text{p}}}_{\text{ij}}\left(1:\text{w}\right)+{\underline{\text{p}}}_{\text{ij}}\left(\text{w}+1:2\text{w}\right)+\ldots +{\underline{\text{p}}}_{\text{ij}}\left(\text{nw}-\text{w}+1:\text{nw}\right)\right]$$

where the underline denotes a vector quantity and n is computed as in Eq. 1. The power P_ij _(w) of each e_ij _(w) is then calculated. The entire procedure is repeated for all pixel traces p_ij_. The average of P_ij _(w) for all i and j is the average power in the image P(w) at period w. P(w) is given by:

(11) $$\text{P}\left(\text{w}\right)=1∕{\text{D}}^{2}\text{w}\;\sum_{\text{ij}}\left[{\underline{\text{e}}}_{\text{ij}}\left(\text{w}\right)\cdot {\underline{\text{e}}}_{\text{ij}}\left(\text{w}\right)\right]$$

where D is the image dimension (= 576), '·' denotes the inner product of the two vectors and i = 1 to n, j = 1 to n. The power computed by Eq. 11 is not equivalent to averaging the grayscale level of all pixels in an image and computing the spectrum from the ensemble averages of mean image grayscale level. That is:

(12) $$\text{P}\left(\text{w}\right)=1∕{\text{D}}^{2}\text{w}\sum_{\text{ij}}\left[{\underline{\text{e}}}_{\text{ij}}\left(\text{w}\right)\cdot {\underline{\text{e}}}_{\text{ij}}\left(\text{w}\right)\right]\neq 1∕{\text{D}}^{2}\text{w}\sum_{\text{ij}}{\underline{\text{e}}}_{\text{ij}}\left(\text{w}\right)\cdot \sum_{\text{ij}}{\underline{\text{e}}}_{\text{ij}}\left(\text{w}\right)$$

The average power P(w) is then multiplied by √n and plotted versus frequency f, as given in Eq. 2. The √n term is a function of w and is computed using Eq. 1. It partially levels the spectral baseline by accounting for the falloff in random noise power as the number of summations n increases. Lastly, linear regression was used to further level the spectral baseline. The DF was selected as the tallest peak in the range 0.05 - 0.67Hz for both Fourier and ensemble power spectra.

## Results

### A. Effect of Spatiotemporal Phase Shift and Random White Noise on Image Series

**Figure 1:**
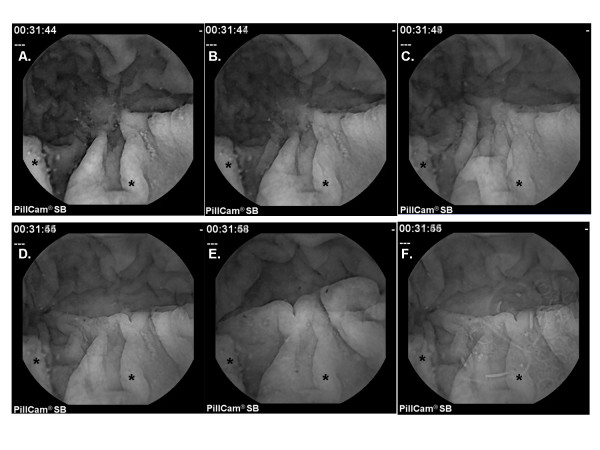
**Videocapsule simulation with random and temporal phase noise and air bubbles imposed**. A. Average of frames 1, 11, ..., 191 before adding phase noise. B-F. Average of frames 1, 11, ..., 191 when phase noise is added to the entire 200 frame series. The temporal phase shift is ± 1, 2, 3, 4, and 5 image frames in panels B-F respectively. In panel F, air bubbles are also imposed.

To show how spatiotemporal phase shift and random white noise affect image sequences, results from celiac patient 1 (w = 10, Table 1) with selected image degradation is shown in Figure 1. In each panel, the result of ensemble averaging of every 10^th ^frame beginning with frame 1 (average of frames 1, 11, ..., 191) is depicted. In other words, the first element of the ensemble average e_ij _(10) as given by Eq. 10 is plotted for all pixels i and j. Panel A shows the average without image degradation. Numerous mucosal folds, small mucosal surface abnormalities, and extraneous substances are evident, as is typical in celiac videoclips obtained from the distal duodenum. A random temporal shift of ± 1 to ± 5 frames was then imposed and shown in panels 1B to 1F, respectively. As a result of the temporal phase shift, the images particularly in panels 1D to 1F increasingly morph to resemble other images that were present in the ten frame series. However, features from the original image having marked contrast as compared to the background tend to be retained - for example as noted by the asterisks in each frame. Additionally, the result after imposition of air bubble frames is shown in panel F. Faint air bubbles are evident within the image, particularly near the center. Spectral analysis of the series would be expected to readily detect the synthetically-derived ten frame period without image degradation being present (panel A), but as progressive degradation occurs (panels 1B through 1F), correct spectral representation would be anticipated to become more challenging.

**Figure 2:**
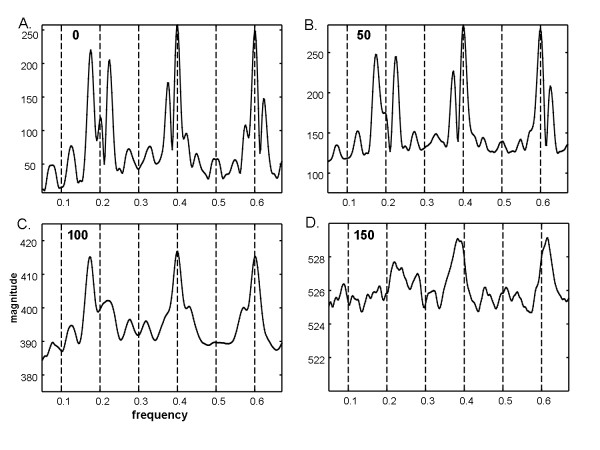
**Fourier power spectra when random and temporal phase noise is imposed**. The random noise magnitude is indicated at top left in each panel and the phase noise is the same for each panel. The actual DF is 0.2 Hz (DP = 5s). However, this peak is split in panels A, B, and C and is eliminated altogether in panel D.

**Figure 3:**
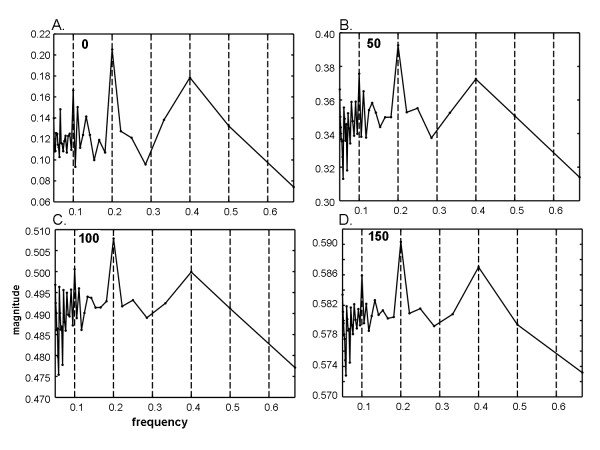
**Ensemble power spectra random and temporal phase noise is imposed as in Figure 2**. In each ensemble power spectrum, the dominant frequency at 0.2Hz (DP = 5s) is the highest peak. As compared with the Fourier power spectra in Figure 2, the ensemble power spectra have no split peaks (panels A, B, and C) and are not degraded by high noise levels (panel D). Unlike for the Fourier power spectra of Figure 2, random and temporal phase noise at these levels have little effect on any of the details in the ensemble power spectra.

For the same series as shown in Figure 1, image degradation of types 1 and 3 described in the Methods section were then added (temporal phase shift of up to ± 4 frames and random noise for × = 0 to 150 frames. The Fourier power spectra are shown in Figure 2, with the random noise level given at top left. The fundamental frequency at 0.2Hz is not very evident as there is a split (triplet) and at the 0.4Hz and 0.6Hz harmonics. Several split harmonic peaks have greater energy than the actual DF at 0.2Hz. As more random white noise frames are added (top left in each panel) the triplets diminish in amplitude and merge (1C to 1D). In panel 1D, the actual DF at 0.2Hz is unrecognizable. Analysis of the same data and noise levels using ensemble averaging is shown in Figure 3. In each panel a dominant peak at 0.2Hz is evident. Subharmonics and the superharmonic at 0.4Hz are present but lesser in value. There are no split peaks. Because of the f = 1/w relationship, the frequency resolution is not uniform (see Methods); however detection of the DF is unaffected.

**Figure 4:**
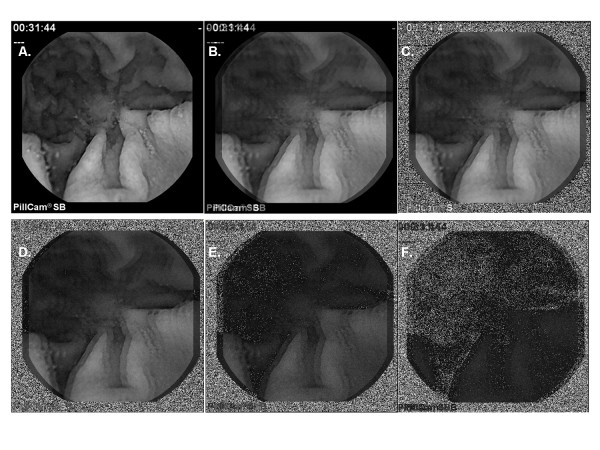
**Videocapsule simulation with random noise and spatial phase shift (jitter) imposed**. A. Average of frames 1, 11, ..., 191 before adding random noise and jitter. B-F. Average of frames 1, 11, ..., 191 when spatial frame shift is used to impart jitter. In all panels B-F, jitter is caused by rotation of each row of pixels by 20 pixels, White noise was present in 0, 25, 50, 75, and 90% of frames used to construct panels B-F, respectively.

Examples when image degradation types 2 and 3 (see Methods) are imposed are depicted in Figure 4 using the same videoclip series as in Figure 1 for ease of comparison (celiac patient 1, Table 1). Panel 4A shows the result when there is no image degradation (same as Figure 1A). Spatial phase noise was added to panels 4B to 4F by imposing a maximum rotation of 20 pixels to the 20 summed images. Additionally, successively increased white noise content, with × = 10, 50, 100, 150, and 180 noise frames substituted at the end of the sequence, are provided in panels 4B to 4F respectively. Figure 4F only slightly resembles the original image (Figure 4A).

**Figure 5:**
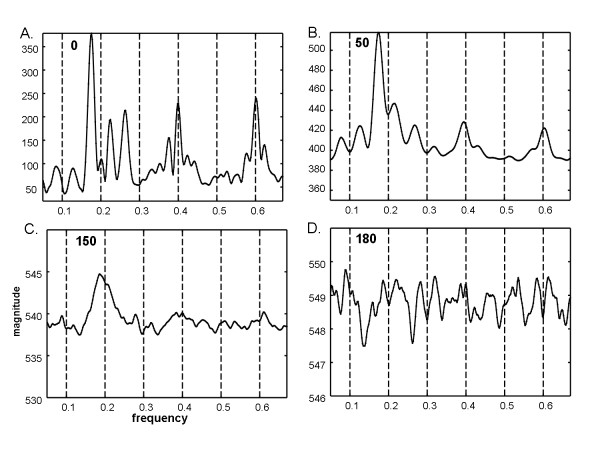
**Fourier power spectra of the average of frames 1, 11, ..., 191 when spatial phase shift, imposed frames with air bubbles, and additive random noise in 0, 25, 75, and 90% of frames in panels A, B, C, and D, respectively**. The actual DF is 0.2 Hz (DP = 5s). As in the Fourier power spectra of Figure 2, split peaks occur about the dominant frequency (panels A and B), and spectral peaks are completely absent at the highest white noise level (panel D). Although there is a peak near the actual dominant frequency in panel C, it is blunted and shifted.

**Figure 6:**
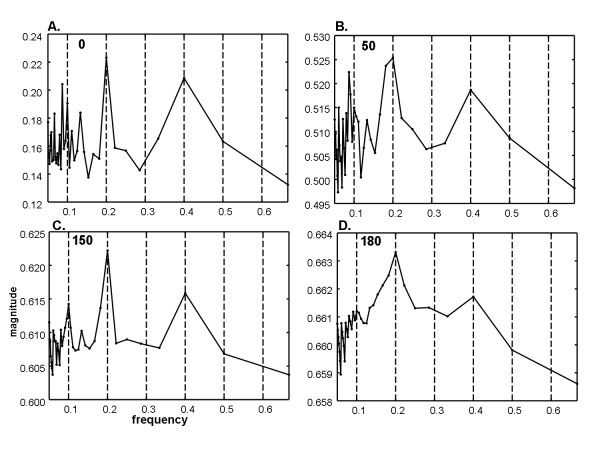
**Ensemble power spectra for same noise levels as in Figure 5**. In each panel the tallest peak is coincident with the actual dominant frequency at 0.2Hz. Most of the other details of the spectra are unchanged from one spectrum to the next even though there is increasing phase and random noise added.

Fourier spectra of the sequence with period w = 10 were also generated when all four image types of degradation were done (see Methods): temporal phase noise, spatial phase noise, additive random noise (value at upper left in each panel), and air bubble frames are shown in Figure 5. Random noise up to 180 frames (panel 5D) was used to show the dramatic change in the spectrum at this level. At lower additive noise levels (Figure 5A and 5B) the harmonic peaks are split as in Figure 2. At higher random noise levels the split peaks merge, but the DF becomes unrecognizable (5D). Analysis of the same data and noise levels using ensemble averaging is shown in Figure 6. In each panel a dominant peak at 0.2Hz is evident. Subharmonics and the superharmonic at 0.4Hz are present but lesser in value than the fundamental. There are no split peaks.

### B. Summary Statistics

In Table 2 the results of tests to detect the DF at four noise levels × = 0, 50, 100, and 150 (see Methods) are shown. The rows indicate patient number, the DP and DF of the simulation, the Fourier estimate of DF with added random noise frames × from 0 to 150, the mean and standard deviation in the Fourier estimation, the difference between the mean Fourier estimate and actual DF, and the mean and standard deviation in the ensemble average estimation. Due to the presence of split peaks, diminished peak height, and noise floor encroachment, only one of the Fourier estimates in Table 2 is precisely correct. In two cases (patients 5 and 6) the second Fourier harmonic was large and the fundamental frequency was nonexistent. The absolute difference (i.e. error) between the mean Fourier estimate and the actual frequency value (F dif) was 0.032 ± 0.052Hz which on the average is a 21% difference when using 0.15Hz as a mean frequency (Table 1). In contrast, all of the ensemble average estimates correctly predicted the actual DF of the simulation thus the standard deviation was zero and the error (E dif) was also zero.

**Table 2:** Summary Statistics of DF Measurement

Pat. #	DP	DF	X = 0	X = 50	X = 100	X = 150	Fourier MN	F dif	Ensemble MN	E dif
**1**	10	0.200	0.176	0.174	0.174	0.186	0.178 ± 0.006	0.022	0.200 ± 0.0	0.0

**2**	11	0.180	0.174	0.172	0.172	0.184	0.176 ± 0.006	0.004	0.180 ± 0.0	0.0

**3**	12	0.167	0.174	0.166	0.178	0.174	0.173 ± 0.005	0.001	0.167 ± 0.0	0.0

**4**	13	0.154	0.174	0.174	0.168	0.174	0.172 ± 0.003	0.002	0.154 ± 0.0	0.0

**5**	14	0.143	0.266	0.266	0.266	0.266	0.266 ± 0.000	0.123	0.143 ± 0.0	0.0

**6**	15	0.133	0.252	0.254	0.252	0.277	0.259 ± 0.012	0.144	0.133 ± 0.0	0.0

**7**	16	0.125	0.129	0.131	0.133	0.119	0.128 ± 0.006	0.006	0.125 ± 0.0	0.0

**8**	17	0.118	0.121	0.123	0.125	0.117	0.122 ± 0.003	0.001	0.118 ± 0.0	0.0

**9**	18	0.111	0.115	0.117	0.115	0.106	0.113 ± 0.005	0.002	0.111 ± 0.0	0.0

**10**	19	0.105	0.109	0.111	0.174	0.184	0.145 ± 0.040	0.040	0.105 ± 0.0	0.0

**11**	20	0.100	0.106	0.106	0.100	0.090	0.101 ± 0.008	0.001	0.100 ± 0.0	0.0

**MN**	--	--	0.163	0.163	0.169	0.171	--	0.032	--	0.0

**SD**	--	--	0.055	0.055	0.052	0.061	--	0.052	--	0.0

DP - actual dominant period in units of number of frames.

DF - actual dominant frequency in units of Hz.

Fourier - the Fourier spectral estimate of DF.

MN = mean and standard deviation

F dif - absolute difference between mean Fourier estimate of DF and the actual frequency value.

E - the ensemble average spectral estimate of DF.

E dif - absolute difference between mean ensemble estimate of DF and the actual frequency value.

X = number of noise frames imposed on the series.

## Discussion

### A. Synopsi*s*

In this study, pixel-by-pixel spectral analysis was introduced for videocapsule image quantification of the small intestine. It was shown that when spatiotemporal phase noise, random white noise, and air bubbles are imposed on videocapsule series acquired from the distal duodenum of celiac patients (Figures 1 and 4), spectral analysis using the ensemble averaging method was useful to detect the dominant frequency (Figures 3 and 6). In contrast, the Fourier method suffered from split peaks (Figures 2A, B, and 2C and 5A, B and 5C) as well as a diminishment of peak height to levels encroaching the noise floor (Figures 2D and 5D). These simulations suggest that the ensemble averaging approach can detect actual periodicities in videocapsule image series more robustly as compared with the Fourier method, thus reducing the need for complex masking, extraction, and/or corrective preprocessing measures. Since prior results suggest a relationship between long periodicity and regions of villous atrophy [8], accurate measurement of the dominant periodicity will be important in future efforts to correlate these parameters to location in the small intestine, and to determine their actual relationship to small intestinal motility.

### B. Analysis of Videocapsule Endoscopy Images

Endoscopy of the small intestine is used for detecting villous atrophy, a common manifestation of untreated celiac disease, which is confirmed by analysis of biopsy specimens acquired during the procedure [20]. Currently, the only treatment for celiac disease patients that can restore the intestinal villi to the healthy state and also eliminate systemic symptoms is a lifelong gluten-free diet [2,20]. Yet, months on the diet are needed to completely restore the villi, and in some patients only partial restoration occurs, or there may be no restoration at all. In a prior quantitative study in which villous atrophy was detected, duodenal features were classified using Fourier filters and magnifying endoscopic images [21]. The method is useful for quantitation except in regions lacking visible change where villous atrophy is still present. Our group has investigated textural properties of images from the small intestinal mucosa in celiac disease [22]. The variation in grayscale brightness can be used as an estimate of texture. It was found over 200 image sequences that celiacs had significantly greater texture even in distal portions of the small intestine (jejunum and ileum) as compared with controls. This suggests the possibility that villous atrophy is widespread in the intestinal lumen in untreated celiac patients, but may be below threshold for visual detection. More recently, frequency analysis was proposed to detect periodic oscillations in frame-to-frame brightness variation that may be related to small intestinal motility [8]. However ambient conditions such as camera angle and illumination may have affected the measurements, since average image brightness level, rather than a pixel-by-pixel analysis, was used to generate the frequency spectrum [8]. By using pixel-level detail to generate spectra, as in the present study, robustness to extraneous influences is likely enhanced, because periodically occurring image features that are spatially related are not masked by the averaging process.

Although our studies thus far have been limited, for simplicity, to converting color videocapsule images to 256-level grayscale for quantitative analysis, abnormal patterns can also be detected in color space using nonlinear methods [23]. It was shown that the green component of RGB color contains much of the detail for detection of small intestinal lesions of the mucosa. Use of a specific color (green, red, or blue) rather than grayscale may therefore be efficacious to improve detection of periodicities with our algorithm, the subject of future work. This study was a first step toward the ultimate goal of the development of computerized quantitative videocapsule analysis that will be used in real time. To meet this goal, any quantitative algorithm must be loaded to a dedicated computer console where videocapsule clips are displayed for analysis by the gastroenterologist. The output would be a frequency spectrum that would be updated in real time with the dominant frequency and period also shown numerically on the graph. A program such as Live Graph (Version 1.1, 2008), which is a real-time data graph plotter, should be useful for this purpose as subsequent versions of our algorithm are developed.

### C. Motility Measurement in Videocapsule Endoscopy

Although videocapsule endoscopy has been commercially available for approximately 10 years, and devices are obtainable from different manufacturers [16,24], the approach is currently used by gastroenterologists as a qualitative assist device when assessing the extent and severity of villous atrophy in celiac patients [25-27]. Gastrointestinal motility is likely altered in untreated celiac disease due to mucosal injury, but this is currently gauged by indirect means i.e., by measuring the transit time from proximal to distal small intestine [1,2]. Other groups have detected specific patterns in videocapsule images and used supervised learning methods to assess intestinal motility and dysfunction in motility [28,29]. In a prior study, correlation between transit time and DP has been shown [8]. Yet, to show that these measurements are actually related to gastrointestinal motility will require external imaging of the small intestine during videocapsule transit, or alternatively, locational information can be provided by a transponder embedded in the videocapsule. Initial investigation of this relationship can be done using an animal model.

### D. Limitations

A limited number of videocapsule series was used to test the methods. Verification should be done in a larger cohort. The methodology supposes, to a first approximation, that camera angle and distance to the mucosal surface is uniform, and that coverage of the surface area of the small intestinal lumen is relatively constant during transit of the videocapsule. However, significant variation in these parameters during capsule transit will likely occur, a limitation of the study.

## Conclusions

In this study it was shown that periodicity in the range 0.1 - 0.2 Hz in videocapsule image sequences can be identified by ensemble averaging spectral analysis even when high levels of random noise, spatiotemporal phase shift, and air bubbles are present. The data was analyzed by constructing a power spectrum for each pixel location over 200 image frames, and averaging, so that repeating spatiotemporal patterns could be identified. By comparison, Fourier power spectra of the same data contained split and missing frequency components, with inaccuracies in DF estimation averaging over 20%. Use of the ensemble method and pixel-level analysis can therefore potentially reduce or eliminate the need to impose complex masking, extraction, or corrective preprocessing measures prior to DF calculation. In future manifestations of the algorithm, we will test the hypothesis that abnormal dominant periodicity as measured from videocapsule image sequences is correlated to motility disorder and to the presence of villous atrophy in celiac patients.

## Competing interests

The authors declare that they have no competing interests.

## Authors' contributions

EJC developed the mathematical method and conducted the data analysis. CAT, GB, SKL, and PHRG provided the clinical data and made helpful suggestions to improve the manuscript. All authors have read and approved the final manuscript.

## References

[B1] Green PH Mortality in celiac disease, intestinal inflammation, and gluten sensitivity JAMA 2009 302 1225 1226 10.1001/jama.2009.1366 19755704

[B2] Tennyson CA Lewis SK Green PH New and developing therapies for celiac disease Therap Adv Gastroenterol 2009 2 303 309 10.1177/1756283X09342759 21180558 PMC3002532

[B3] Ludvigsson JF Green PH Clinical management of coeliac disease J Intern Med 2011 269 6 560 571 10.1111/j.1365-2796.2011.02379.x 21443532

[B4] Rey JF Repici A Kuznetsov K Boyko V Aabakken L Optimal preparation for small bowel examinations with video capsule endoscopy Dig Liver Dis 2009 41 486 493 10.1016/j.dld.2008.09.016 19158002

[B5] Triantafyllou K Can we improve the diagnostic yield of small bowel video-capsule endoscopy? World J Gastrointest Endosc 2010 2 143 146 10.4240/wjgs.v2.i4.143 21160741 PMC2999125

[B6] Vilarino F Spyridonos P Pujol O Vitria J Radeva P DeIorio F Automatic Detection of Intestinal Juices in Wireless Capsule Video Endoscopy Pattern Recognition 2006 2006 4 18th International Conference, Hong Kong; FCPR 719 722

[B7] Vilarino F Spyridonos P DeIorio F Vitria J Azpiroz F Radeva P Intestinal motility assessment with video capsule endoscopy: automatic annotation of phasic intestinal contractions IEEE Trans Med Imaging 2010 29 246 259 10.1109/TMI.2009.2020753 19423434

[B8] Ciaccio EJ Tennyson CA Lewis SK Krishnareddy S Bhagat G Green PHR Distinguishing patients with celiac disease by quantitative analysis of videocapsule endoscopy images Comput Methods Programs Biomed 2010 100 1 39 48 10.1016/j.cmpb.2010.02.005 20356648

[B9] Botteron GW Smith JM A technique for measurement of the extent of spatial organization of atrial activation during atrial fibrillation in the intact human heart IEEE Trans Biomed Eng 1995 42 579 586 10.1109/10.387197 7790014

[B10] Botteron GW Smith JM Quantitative assessment of the spatial organization of atrial fibrillation in the intact human heart Circulation 1996 93 513 518 8565169 10.1161/01.cir.93.3.513

[B11] Fischer G Stühlinger MC Nowak CN Wieser L Tilg B Hintringer F On computing dominant frequency from bipolar intracardiac electrograms IEEE Trans Biomed Eng 2007 54 165 169 17260870 10.1109/TBME.2006.883739

[B12] Ng J Kadish AH Goldberger JJ Effect of electrogram characteristics on the relationship of dominant frequency to atrial activation rate in atrial fibrillation Heart Rhythm 2006 3 1295 1305 10.1016/j.hrthm.2006.07.027 17074635

[B13] Ng J Kadish AH Goldberger JJ Technical considerations for dominant frequency analysis J Cardiovasc Electrophysiol 2007 18 757 764 10.1111/j.1540-8167.2007.00810.x 17578346

[B14] Ciaccio EJ Biviano AB Whang W Wit AL Garan H Coromilas J New methods for estimating local electrical activation rate during atrial fibrillation Heart Rhythm 2009 6 21 32 10.1016/j.hrthm.2008.10.016 19121796

[B15] Ciaccio EJ Biviano AB Whang W Wit AL Coromilas J Garan H Optimized measurement of activation rate at left atrial sites with complex fractionated electrograms during atrial fibrillation J Cardiovasc Electrophysiol 2010 21 133 143 10.1111/j.1540-8167.2009.01595.x 19793138

[B16] Metzger YC Adler SN Shitrit AB Koslowsky B Bjarnason I Comparison of a new PillCam™ SB2 video capsule versus the standard PillCam™ SB for detection of small bowel disease Reports in Medical Imaging 2009 2 7 11

[B17] Ciaccio EJ Biviano AB Whang W Coromilas J Garan H A new transform for the analysis of complex fractionated atrial electrograms Biomed Eng Online 2011 10 35 10.1186/1475-925X-10-35 21569421 PMC3125385

[B18] Press WH Flannery BP Teukolsky SA Vetterling WT Numerical Recipes in C: The Art of Scientific Computing 1992 2 New York, NY: Cambridge Univ. Press 501 502

[B19] Frigo M Johnson SG The design and implementation of FFTW3 Proc IEEE 2005 93 216 231

[B20] Green PH Celiac disease: how many biopsies for diagnosis? Gastrointest Endosc 2008 67 1088 1090 10.1016/j.gie.2007.12.035 18513550

[B21] Vécsei A Fuhrmann T Liedlgruber M Brunauer L Payer H Uhl A Automated classification of duodenal imagery in celiac disease using evolved Fourier feature vectors Comput Methods Programs Biomed 2009 95 2 Suppl S68 S78 19356823 10.1016/j.cmpb.2009.02.017

[B22] Ciaccio EJ Tennyson CA Bhagat G Lewis SK Green PH Classification of videocapsule endoscopy image patterns: comparative analysis between patients with celiac disease and normal individuals BioMed Eng OnLine 2010 9 44 55 10.1186/1475-925X-9-44 20815911 PMC2941491

[B23] Charisis V Hadjileontiadis LJ Liatsos CN Mavrogiannis CC Sergiadis GD Abnormal pattern detection in wireless capsule endoscopy images using nonlinear analysis in RGB color space Conf Proc IEEE Eng Med Biol Soc 2010 1 Engineering in Medicine and Biology Society (EMBC), 2010 Annual International Conference of the IEEE Aug. 31 2010-Sept. 4 2010 Buenos Aires 3674 3677 10.1109/IEMBS.2010.5627648 21097046

[B24] Gheorghe C Iacob R Bancila I Olympus capsule endoscopy for small bowel examination J Gastrointestin Liver Dis 2007 16 309 313 17925927

[B25] Culliford A Daly J Diamond B Rubin M Green PH The value of wireless capsule endoscopy in patients with complicated celiac disease Gastrointest Endosc 2005 62 55 61 10.1016/S0016-5107(05)01566-X 15990820

[B26] Green PH Rubin M Capsule endoscopy in celiac disease Gastrointest Endosc 2005 62 797 799 10.1016/j.gie.2005.07.034 16246703

[B27] Green PH Rubin M Capsule endoscopy in celiac disease: diagnosis and management (Review) Gastrointest Endosc Clin N Am 2006 16 307 316 10.1016/j.giec.2006.03.003 16644459

[B28] Seguí S Igual L Radeva P Malagelada C Azpiroz F Vitrià J A semi-supervised learning method for motility disease diagnostic Progress in Pattern Recognition, Image Analysis and Applications Lecture Notes in Computer Science 2007 4756 773 782

[B29] Seguí S Igual L Vilariño F Radeva P Malagelada C Azpiroz F Vitrià J Diagnostic system for intestinal motility disfunctions using video capsule endoscopy Computer Vision Systems Lecture Notes in Computer Science 2008 5008 251 260 10.1007/978-3-540-79547-6_24

